# *Defluviitalea raffinosedens* sp. nov., a thermophilic, anaerobic, saccharolytic bacterium isolated from an anaerobic batch digester treating animal manure and rice straw

**DOI:** 10.1099/ijsem.0.001664

**Published:** 2017-05-09

**Authors:** Shichun Ma, Yan Huang, Cong Wang, Hui Fan, Lirong Dai, Zheng Zhou, Xing Liu, Yu Deng

**Affiliations:** ^1^​ Biogas Institute of Ministry of Agriculture, Chengdu, Sichuan, P.R. China; ^2^​ Key Laboratory of Energy Microbiology and Application, Ministry of Agriculture, Chengdu, Sichuan, P.R. China; ^3^​ College of Light Industry, Textile and Food Engineering, Sichuan University, Chengdu, Sichuan, P.R. China; ^‡^​ Present address: Biogas Institute of Ministry of Agriculture, Section 4-13, Renmin Nan Road, Chengdu 610041, Sichuan, P.R. China.; ^§^​ Present address: College of Light Industry, Textile and Food Engineering, Sichuan University, No.24 South Section 1, Yihuan Road, Chengdu 610065, Sichuan, P.R. China.

**Keywords:** thermophilic, saccharolytic, *Defluviitalea raffinosedens*, *Defluviitaleaceae*

## Abstract

A thermophilic, anaerobic, fermentative bacterium, strain A6^T^, was obtained from an anaerobic batch digester treating animal manure and rice straw. Cells were Gram-stain-positive, slightly curved rods with a size of 0.6–1×2.5–8.2 µm, non-motile and produced terminal spores. The temperature, pH and NaCl concentration ranges for growth were 40–60 °C, 6.5–8.0 and 0–15.0 g l^−1^, with optimum growth noted at 50–55 °C, pH 7.5 and in the absence of NaCl, respectively. Yeast extract was required for growth. d-Glucose, maltose, d-xylose, d-galactose, d-fructose, d-ribose, lactose, raffinose, sucrose, d-arabinose, cellobiose, d-mannose and yeast extract were used as carbon and energy sources. The fermentation products from glucose were ethanol, lactate, acetate, propionate, butyrate, valerate, iso-butyrate, iso-valerate, H_2_ and CO_2_. The G+C content of the genomic DNA was 36.6 mol%. The predominant fatty acids were C_16 : 0_, iso-C_17 : 1_, C_14 : 0_, C_16 : 1_*ω*7*c*, C_16 : 0_ N-alcohol and C_13 : 0_ 3-OH. Respiratory quinones were not detected. The polar lipid profile comprised phosphoglycolipids, phospholipids, glycolipids, a diphosphatidylglycerol, a phosphatidylglycerol and an unidentified lipid. Phylogenetic analyses of the 16S rRNA gene sequence indicated that the strain was closely related to *Defluviitalea saccharophila* DSM 22681^T^ with a similarity of 96.0 %. Based on the morphological, physiological and taxonomic characterization, strain A6^T^ is considered to represent a novel species of the genus *Defluviitalea*, for which the name *Defluviitalea raffinosedens* sp. nov. is proposed. The type strain is A6^T^ (=DSM 28090^T^=ACCC 19951^T^).

Anaerobic digestion is a method of waste treatment aimed at reducing the hazardous effects of wastes on the biosphere [1]. It comprises complex, redox biochemical reactions driven by various anaerobic and relatively anaerobic micro-organisms, resulting in the decomposition of complex organic substances into simple compounds (mainly CH_4_ and CO_2_) [2]. Since the beginning of the use of culture-independent techniques, increasing numbers of ecological studies have indicated that the phylum *Firmicutes* is one of the predominant and widespread bacterial groups in various anaerobic digesters [3–6]. It is well known that groups of the order *Clostridiales* in the phylum *Firmicutes* (such as *Clostridium*, *Acetivibrio*, *Selenomonas* and *Ruminococcus*) are some of the most common hydrolytic bacteria in anaerobic bioreactors, especially in cellulolytic environments [7–11]. *Defluviitaleaceae*, belonging to the order *Clostridiales* of the phylum *Firmicutes*, was erected by Jabari [12] to describe thermophilic, anaerobic, Gram-positive, rod-shaped, non-motile, terminal-spore-forming and saccharolytic bacteria. *Defluviitalea saccharophila* LIND6LT2^T^ was isolated from an upflow anaerobic digester treating waste water, and was assigned as the type species of the family *Defluviitaleaceae*.

We collected samples from an anaerobic batch digester treating animal manure and rice straw, which was pre-enriched with PY medium (2 g peptone and 1 g yeast extract per litre distilled water) containing rice straw (5 g per litre distilled water), and a microbial consortium degrading rice straw under anaerobic methanogenic conditions at 40 °C was obtained and subcultured for 10 years. The 16S rRNA clone libraries and high-throughput sequencing analyses revealed that *Clostridium*, *Gracilibacter, Sedimentibacter* and uncultured *Firmicutes* were the predominant organisms of the microbial consortium (unpublished data). To reveal the ecophysiological roles of anaerobic bacteria in anaerobic digestion, strain A6^T^ was enriched and isolated from the above-mentioned microbial consortium at 55 °C using enriched medium (basal medium containing 1 g yeast extract and 5 g sodium acetate or 3 g sodium propionate). The basal medium contained the following (per litre distilled water): NH_4_Cl, 1.0 g; yeast extract, 0.1 g; l-Cys-HCl, 1 g; 0.1 % (w/v) resazurin solution, 1.0 ml; macro mineral solution, 50.0 ml; trace mineral solution, 10.0 ml; and vitamin mix solution, 10.0 ml. The macro mineral solution, trace mineral solution and vitamin mix solution were prepared as described previously [13]. The agar medium was supplemented with 18.0 g agar. All the media were prepared and dispensed anaerobically under a gaseous atmosphere of 100 % N_2_. The pH of the medium was adjusted to 6.5–7.0 with 5 M KOH, and the media were sterilized by autoclaving at 121 °C for 30 min.

The enriched medium was inoculated with 2 % (v/v) rice-straw-degrading microbial consortium and incubated for 1 week at 55 °C. For isolation, the enrichment culture was serially diluted tenfold in Hungate tubes containing molten agar medium, and the tubes were rolled following the procedures of the Hungate roll-tube technique [14–16]. Subsequently, single colonies were picked and transferred into liquid medium under anaerobic conditions. The roll-tube procedure was repeated several times until a pure culture was obtained. A single white and round colony was obtained and designated as strain A6^T^. This strain did not utilize acetate or propionate, but grew at low cell concentration in enriched medium, indicating that the yeast extract in the medium served as carbon and energy source during enrichment and isolation. For subsequent incubation of strain A6^T^, d-glucose was used as the main substrate, instead of acetate or propionate. The taxonomic description of strain A6^T^ is reported here based on phenotypic and phylogenetic studies.

The extraction and purification of DNA, PCR amplification and sequencing of the 16S rRNA were performed as described by Huang [17]. The sequence obtained was submitted to NCBI for initial alignment with highly similar sequences in the blastn program. The 16S rRNA sequences from closely related organisms were retrieved from NCBI and EzTaxon. Phylogenetic trees were reconstructed with the software package mega version 5.0 using the neighbour-joining and maximum-likelihood methods [18]. The robustness of the topology in the phylogenetic tree was evaluated by bootstrap analysis based on 1000 replicates. Phylogenetic analysis based on the 16S rRNA gene sequence revealed that strain A6^T^ belonged to the family *Defluviitaleaceae*, and that its closest relative was *D. saccharophila* LIND6LT2^T^ (96 % sequence similarity), followed by *Natranaerovirga pectinivora* AP3^T^ (88.9 %), *Vallitalea guaymasensis* Ra1766G1^T^ (88.4 %), *Lactonifactor longoviformis* ED-Mt61/PYG-s6^T^ (88.3 %) and *Anaerostipes butyraticus* LMG 24724^T^ (88.11 %) (Fig. 1).

**Fig. 1.**
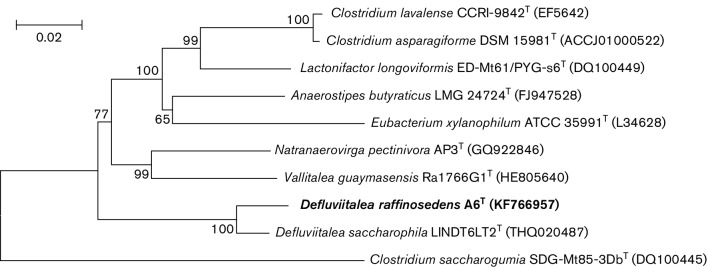
Neighbour-joining phylogenetic tree based on 16S rRNA gene sequences showing the relationship between strain A6^T^ and its phylogenetically close relatives. The GenBank/EMBL/DDBJ accession number for the 16S rRNA gene sequence of strain A6^T^ is KF766957 (1413 bp). Bar, 0.02 changes per nucleotide position.

The cultural and morphological characteristics of the isolated strain A6^T^ were investigated using cells cultivated on basal carbonate yeast extract and trypticase medium (BCTY medium). The BCTY medium consisted of basal medium, yeast extract (0.5 g l^−1^) and trypticase (0.5 g l^−1^). Prior to inoculation, filter-sterilized glucose solution was added as substrate (final concentration 5 g l^−1^) to the sterile BCTY medium. Cell morphology was examined using a scanning electron microscope (JEOL JSM-7500F) and transmission electron microscope (Hitachi H-600IV). Gram staining was performed using the traditional method [19] and spore staining was performed conventionally [20]. The presence of spores and Gram staining were observed using a phase-contrast microscope (Nikon 80i).

*D. saccharophila* DSM 22681^T^ was obtained from the Deutsche Sammlung von Mikroorganismen und Zellkulturen GmbH (DSMZ) for comparson of its physiological and chemotaxonomic characteristics with those of strain A6^T^. Growth experiments to determine the pH, temperature and NaCl concentration ranges were performed in triplicate using Hungate tubes with 5 ml of BCTY medium containing glucose as the substrate. The pH range examined for growth was 5.5–10.0, and was adjusted using the following sterile anaerobic solutions (20 mM): MES (5.5, 6.0), PIPES (6.5, 7.0, 7.5), HEPES (8.0), Tricine (8.5) and CHES (9.0, 9.5, 10.0). The temperature range investigated was 35–65 °C at 5 °C intervals, and the NaCl concentration range was 0–25.0 g NaCl l^−1^. Substrate utilization tests were performed in basal medium with d-glucose, d-xylose, maltose, d-fructose, d-galactose, d-ribose, d-sucrose, d-lactose, d-mannose, d-mannitol, raffinose, l-rhamnose, cellobiose, d-arabinose, yeast extract, acetate, propionate, pyruvate and lactate. Each substrate was added at a final concentration of 20 mM (for sugars and organic acids). The strain was subcultured at least twice under the same experimental conditions prior to determination of growth rates. Elemental sulfur (1 %, w/v), sulfate (20 mM), thiosulfate (20 mM), sulfite (2 mM), nitrate (10 mM) and nitrite (2 mM) were tested as terminal electron acceptors. Growth was determined by measuring the turbidity of the cultures at a wavelength of 600 nm using a spectrophotometer (DU 730; Beckmann) as described previously [12].

The liquid fermentation products were determined by GC (Agilent 7890A) using an FFAP column (30 m×320 µm×0.25 µm) and a ﬂame ionization detector with N_2_ as the carrier gas at a flow rate of 36 ml min^−1^. H_2_ and CO_2_ were analysed by GC (Agilent 7820A) using a porapak Q packed column (2 m×30 µm) and thermal conductivity detector with N_2_ as the carrier gas at a ﬂow rate of 30.0 ml min^−1^ and column temperature of 80 °C. H_2_S production was determined photometrically as described by Cord-Ruwisch [21]. Sulfate, nitrate and nitrite were measured by ion chromatography (Dionex ICS-3000) using an IonPac AG12A column with 2.7 mM Na_2_CO_3_ and 0.3 mM NaHCO_3_ as eluent at a flow rate of 1.2 ml min^−1^.

Strain A6^T^ formed white and round colonies after 2 days at 55 °C. Cells were non-motile and slightly curved rods with a size of 2.5–7.6×1–0.58 µm, occurring singly or in pairs (Fig. 2). Furthermore, the strain was Gram-stain-positive and formed spores at high temperature. The temperature, pH and NaCl concentration ranges for growth of the strain were 40–65 °C (optimum 50 °C), 6.5–8.0 (optimum 7.5) and 0–20 % (w/v) (optimum 0 %), respectively (Fig. S1, available in the online Supplementary Material). The maximum growth rate of the strain was 0.58 h^−1^ when glucose was used as the substrate in BCTY medium under the above-mentioned optimum conditions.

**Fig. 2.**
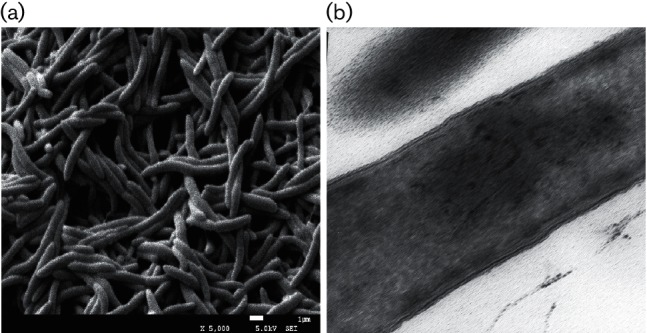
(a) Scanning electron micrograph of cells of strain A6^T^. (b) Transmission electron micrograph of thin sections of cells cultured for 24 h. Bars, 1 µm (a), 0.5 µm (b).

It is noteworthy that the addition of yeast extract enhanced growth of strain A6^T^. Similar to *D. saccharophila* LIND6LT2^T^, growth of strain A6^T^ was improved with increasing concentrations of yeast extract. Elemental sulfur, thiosulfate, sulfite, sulfate and nitrate were not used as electron acceptors. While the strain was able to ferment d-glucose, maltose, d-xylose, d-galactose, d-fructose, d-ribose, lactose, raffinose, sucrose, d-arabinose, cellobiose, d-mannose and yeast extract, it could not utilize d-mannitol, l-rhamnose, peptone, acetate, propionate, pyruvate or lactate. Moreover, cellulose was not hydrolysed by strain A6^T^. The fermentation products of the strain in a saccharide-utilizing culture were H_2_, CO_2_, ethanol, lactate, acetate, propionate, butyrate, valerate, traces of iso-butyrate, and iso-valerate.

The DNA G+C content, cellular fatty acid composition, respiratory quinones and polar lipids were evaluated by the Identification Service of the DSMZ (Braunschweig, Germany). The DNA G+C content was determined by using HPLC as described by Mesbah *et al.* [22]. The cellular fatty acid composition was determined by saponification, methylation and extraction as described earlier with minor modifications [23, 24]. Fatty acids were analysed using the Sherlock MIS system (MIDI). Respiratory quinones were extracted using methanol/hexane [25, 26], followed by phase separation into hexane. Respiratory lipoquinones were separated by TLC on silica gel (Macherey-Nagel Art. No. 805023), using hexane/tetrabutylmethylether (9 : 1, v/v) as the solvent and further analysed by HPLC. Polar lipids were extracted using chloroform/methanol/0.3 % aqueous NaCl mixture (1 : 2 : 0.8, by vol.) and separated by two-dimensional silica gel TLC (Macherey-Nagel Art. No. 18135). The total lipid content was detected using the method described by Tindall *et al.* [27].

The major whole-cell fatty acids of strain A6^T^ were C_16 : 0_ (30.6 %), iso-C_17 : 1_ (30.3 %), C_14 : 0_ (18.1 %), C_16 : 1_ω7*c* (5.6 %), C_16 : 0_ N-alcohol (3.2 %), C_13 : 0_ 3-OH (2.9 %), C_13 : 1_ AT 12–13 (1.0 %), C_18 : 0_ (0.8 %), C_12 : 0_ (0.7 %), C_18 : 1_ω7*c* (0.4 %), C_16 : 1_ω5*c* (0.3 %), C_18 : 1_ω9*c* (0.3 %) and an unknown component (5.7 %) (Table 1). Respiratory quinones were not detected. The polar lipid profile comprised phosphoglycolipids, phospholipids, glycolipids, a diphosphatidylglycerol and a phosphatidylglycerol (Fig. S2). The DNA G+C content of strain A6^T^ was 36.6 mol%, which is similar to that of *D. saccharophila* LIND6LT2^T^ (35.2 mol%) [12].

**Table 1.** Comparison of the cellular fatty acid profiles of strain A6^T^ with its phylogenetically closest relative

Fatty acid	Strain A6^T^	*D. saccharophila* LIND6LT2^T^
C_12 : 0_	0.7	0.4
C_13 : 1_ AT 12–13	1.0	–
C_14 : 0_	18.1	8.3
C_13 : 0_ 3-OH	2.9	–
C_16 : 0_	30.6	68.4
C_16 : 0_ N-alcohol	3.2	0.7
C_16 : 1_ω7*c*	5.6	–
C_16 : 1_ω5*c*	0.3	5.3
iso-C_17 : 1_	30.3	–
C_18 : 1_ω9*c*	0.3	0.8
C_18 : 1_ω7*c*	0.4	4.1
C_18 : 0_	0.8	7.3
Unknown	5.7	1.4

Although strain A6^T^ was found to be phenotypically comparable to *D. saccharophila* LIND6LT2^T^ with respect to cell morphology, optimum pH and temperature for growth, electron acceptors, and polar lipid profile, it differed with respect to major cellular fatty acids and substrate utilization. Unlike *D. saccharophila* LIND6LT2^T^, strain A6^T^ did not ferment d-mannitol or l-rhamnose, but fermented d-galactose, d-fructose, d-ribose, lactose, raffinose and d-arabinose (Table 2). Moreover, strain A6^T^ could be easily distinguished from *N. pectinivora*, *V. guaymasensis*, *L. longoviformis* and *A. butyraticus* by growth temperature and DNA G+C content. In addition, strain A6^T^ and *N. pectinivora* could also be differentiated based on the utilization of pectinous substrates.

**Table 2.** Phenotypic comparison of strain A6^T^ with its five phylogenetically closest relatives Taxa: 1, strain A6^T^; 2, *D. saccharophila* [12]; 3, *N. pectinivora* [28]; 4, *V. guaymasensis* [29]; 5, *L. longoviformis* [30]; 6, *A. butyraticus* [31]. +, Positive; −, negative or very weakly positive; nd, not done. APL, aminophospholipid; DPG, diphosphatidylglycerol; GL, glycolipid; PG, phosphatidylglycerol; PGL, phosphoglycolipid; PL, phospholipid; L, unknown lipid; EtOH, ethanol; A, acetate; P, propionate; B, butyrate; iB, isobutyrate; F, formate; V, valerate; iV, isovalerate; L, lactate.

Characteristic	1	2	3	4	5	6
Gram stain	+	+	+	−	+	+
Morphology	Slightly curved rods (1–0.58×2.5–7.6 µm)	Rods (0.5×5–10 µm)	Rods with variable length (0.25–3×3–10 µm)	Rods (0.5–1×2–10 µm)	Rods (1.0–1.5×1.5–3.0 µm)	Long rods (5–15 µm)
Temperature (optimum) (°C)	50	50–55	43 (max.)	30–35	37	37–41
pH	7.5	7–7.5	9.5–9.7	6.5–7.5	5.5–9.3	6
NaCl concentration (%, w/v)	0	0.5	0.4–0.6 M Na^+^	2–3	nd	
Motility	−	−	−	nd	−	nd
Major cellular fatty acids	C_16 : 0_, iso-C_17 : 1_, C_14 : 0_	C_16 : 0_, C_14 : 0_, C_18 : 0_	C_16 : 0_, C_16 : 1_ω7*c*, C_18 : 1_ω7*c*	anteiso-C_15 : 0_, iso-C_15 : 0_, anteiso DMA-C_15 : 0_, C_16 : 0_.	C_16 : 0_	nd
Polar lipids	PGL, PL, GL, DPG, PL, L	DPG, PG, PL, PGL, GL	PG, DPG, PL, GL, APL	DPG, PG, GL, PL	nd	
DNA G+C content (mol%)	36.6	35.2	30.7	31.2	48	44
Substrates						
d-Glucose	+	+	−	+	+	+
d-Xylose	+	+	−	+	−	−
d-Ribose	+	−	−	+	+	nd
d-Arabinose	+	−	−	+	−	−
d-Galactose	+	−	−	+	+	nd
d-Fructose	+	−	−	−	+	nd
Cellobiose	+	+	−	+	−	+
Sucrose	+	+	−	+	+	+
d-Lactose	+	−	−	−	−	−
d-Mannose	+	+	−	+	+	+
Maltose	+	+	−	+	−	+
Raffinose	+	−	−	+	+	+
d-Mannitol	−	+	−	−	−	+
l-Rhamnose	−	+	−	−	−	−
Others			Galacturonic acid, pectin, polygalacturonates	Pyruvate	Sorbitol, melibiose, melezitose	Salicin, sorbitol, trehalose
Fermentation end products	H_2_, CO_2_, EtOH, L, A, P, B, iB, V, iV,	H_2_, CO_2_, A, B, F, iB,	A, F	A	nd	B, A, P, H_2_, CO_2_

Therefore, based on the data from phylogenetic, physiological and chemotaxonomic analyses, strain A6^T^ can be considered to represent a novel species of the genus *Defluviitalea,* belonging to the family *Defluviitaleaceae*, order *Clostridiales*, and phylum *Firmicutes*, for which we propose the name *Defluviitalea raffinosedens* sp. nov.

## Description of *Defluviitalea raffinosedens* sp. nov

*Defluviitalea raffinosedens* (raf.fi.nos.e′dens. N.L. neut. n. *raffinosum* raffinose; L. pres. part. *edens* eating; N.L. part. adj. *raffinosedens* raffinose-eating).

Cells are Gram-stain-positive, slightly curved rods with a size of 2.5–7.6×1–0.58 µm, non-motile, occur singly or in pairs, and form spores at high temperature. Growth occurs at 40–65 °C (optimum 50 °C), pH 6.5–8.0 (optimum 7.5) and an NaCl concentration of 0–20 % (w/v). Cells are thermophilic and anaerobic, and hydrolyse d-glucose, maltose, d-xylose, d-galactose, d-fructose, d-ribose, lactose, d-mannose, raffinose, sucrose, d-arabinose, cellobiose and yeast extract. Yeast extract is required for growth. The major cellular fatty acids are C_16 : 0_, iso-C_17 : 1_, C_14 : 0_, C_16 : 1_ω7*c*, C_16 : 0_ N-alcohol and C_13 : 0_ 3-OH. Respiratory quinones are not found. The polar lipids are phosphoglycolipids, phospholipids, glycolipids, a diphosphatidylglycerol and a phosphatidylglycerol.

The type strain is A6^T^ (=DSM 28090^T^=ACCC 19951^T^), isolated from an anaerobic batch digester treating animal manure and rice straw. The G+C content of the genomic DNA of the type strain is 36.6 mol%.

## Supplementary Data

306 Supplementary File 1
