# Supplementary material for: Defluviitalea raffinosedens sp. nov., a thermophilic, anaerobic, saccharolytic bacterium isolated from an anaerobic batch digester treating animal manure and rice straw
Source: Int J Syst Evol Microbiol. 2017 May 9;67(5):1607–12. doi: 10.1099/ijsem.0.001664 (PMC5817277; doi:10.1099/ijsem.0.001664)
Supplement: Supplementary File 1 [file ijsem-67-1607-s001.pdf]

Supplementary materials

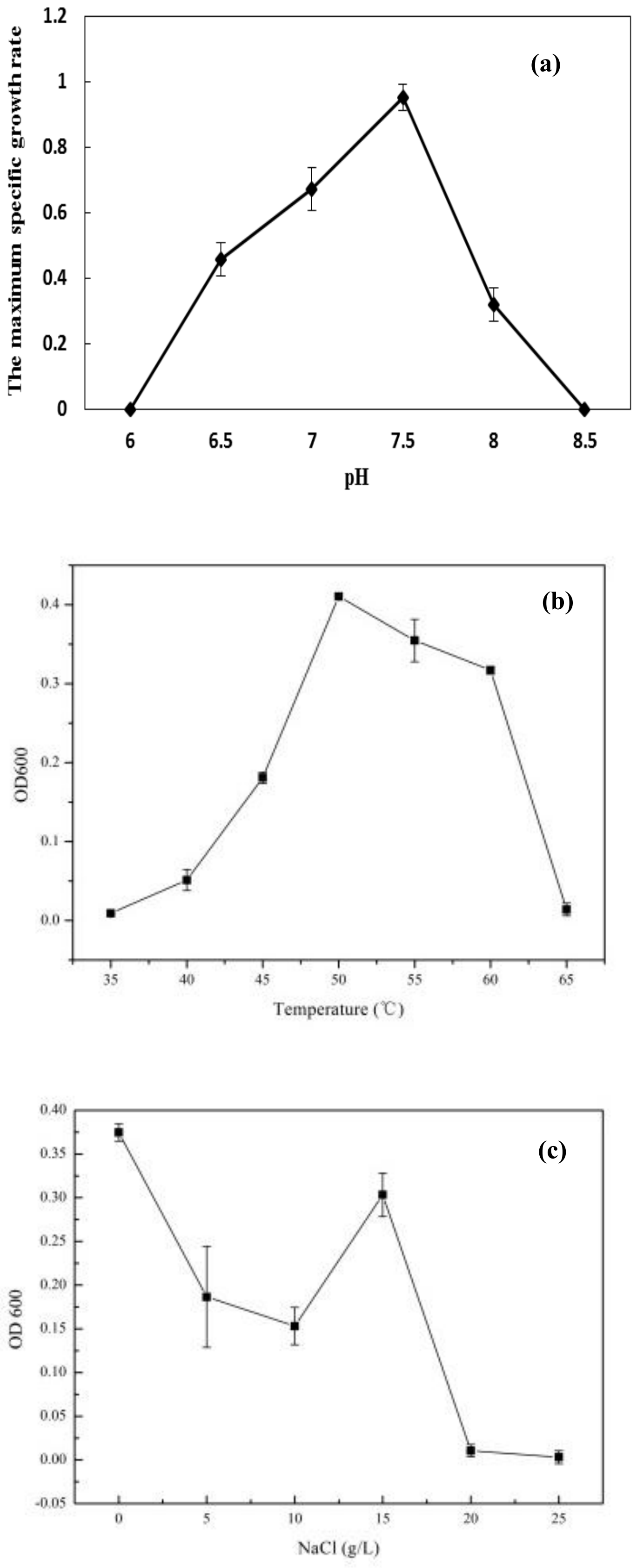

Fig. S1. Effects of pH (a), temperature (b), and NaCl concentration (c) on the growth of strain A6^{T}.

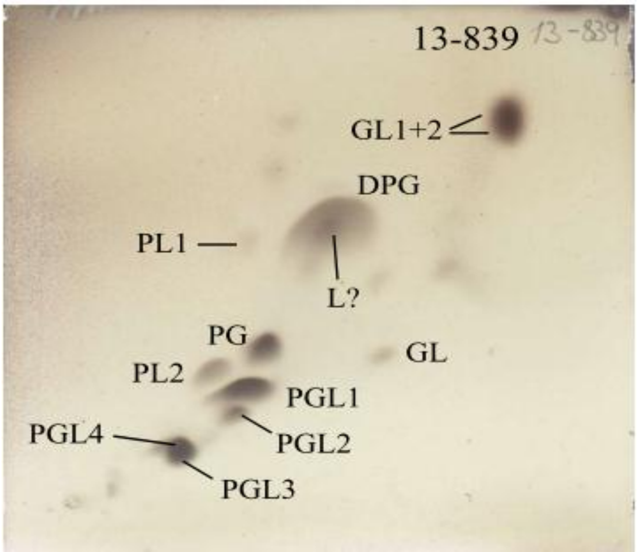

Fig. S2. Polar lipid profile of strain A6^{T}
